# Monitoring Mitochondrial Function With High‐Resolution Respirometry During Ex‐Situ Heart Perfusion

**DOI:** 10.1111/aor.70213

**Published:** 2026-09-10

**Authors:** Lukas Stastny, Julia Hofmann, Andras T. Meszaros, Alexandra Ampferer, Gabriel Putzer, Nina Perkins, Natalie Huemer, Florian Sommerauer, Judith Martini, Julian Wagner, Michael Grimm, Stefan Schneeberger, Julia Dumfarth

**Affiliations:** ^1^ Department of Cardiac Surgery Medical University of Innsbruck Innsbruck Austria; ^2^ Department of Visceral, Transplant and Thoracic Surgery Medical University of Innsbruck Innsbruck Austria; ^3^ Department of Anaesthesiology and Critical Care Medicine Medical University of Innsbruck Innsbruck Austria; ^4^ Department of Cardiac and Thoracic Aortic Surgery Medical University of Vienna Vienna Austria

**Keywords:** ex‐situ heart perfusion, ischemia–reperfusion injury, mitochondrial respiration

## Abstract

**Background:**

Due to the high adenosine‐triphosphate (ATP) consumption of the myocardial tissue, cardiac function is dependent on efficient mitochondrial respiration. Ischemia and reperfusion injury (I/R) has a severe impact on the function of the mitochondrial respiratory system. *Ex‐situ* heart perfusion (ESHP) can minimize I/R injury. We investigated mitochondrial respiration using high‐resolution respirometry (HRR) in a porcine model during ESHP.

**Methods:**

Six German domestic pigs were used as heart and blood donors. Normothermic ESHP was performed over 6 h. Tissue homogenate of porcine myocardium was assessed by HRR at baseline (starting sample), after 60, 180, and 360 min of ESHP.

**Results:**

The combined oxidative phosphorylation capacity with NADH, fatty acid and succinate fuel substrates remained stable during ESHP (starting sample: 198 ± 59.7, 60 min: 196.5 ± 66.7, 180 min: 207.5 ± 48.1, 360 min: 200.5 ± 27.9 pmol·s^−1^·mg wet mass^−1^; *p* = 0.707). The flux control ratio of the nicotinamide adenine dinucleotide hydrogen (NADH) pathway (starting sample: 0.43 ± 0.07, 60 min: 0.43 ± 0.05, 180 min: 0.47 ± 0.05, 360 min: 0.44 ± 0.07 pmol·s^−1^·mg wet mass^−1^; *p* = 0.549) and succinate‐pathway (starting sample: 0.49 ± 0.06, 60 min: 0.49 ± 0.05, 180 min: 0.48 ± 0.01, 360 min: 0.5 ± 0.05 pmol·s^−1^·mg wet mass^−1^; *p* = 0.814) did not change during perfusion. The mitochondrial outer membrane damage was significantly decreased after 360 min of ESHP (starting sample: 0.39 ± 0.07, 60 min: 0.27 ± 0.06, 180 min: 0.18 ± 0.03, 360 min: 0.17 ± 0.02; *p* = 0.0124).

**Conclusion:**

Our findings demonstrate that normothermic ESHP preserves mitochondrial respiratory function in a porcine model over 6 h. These results provide a mechanistic basis for the potential protective role of normothermic ESHP against ischemia–reperfusion injury. HRR represents a promising investigational tool for in‐depth mitochondrial assessment during ESHP.

## Graphical abstract

Normothermic ESHP preserves mitochondrial respiratory function over 6 h in a porcine model (*n* = 6): OXPHOS capacities across NADH‐, fatty‐acid‐, and succinate‐linked pathways remain stable, with reduced cytochrome‐c control efficiency indicating improved outer‐membrane integrity. HRR provides a rapid, actionable readout for organ assessment.

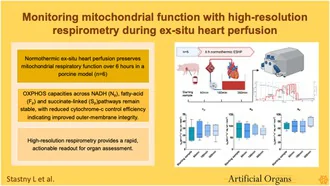

## 1 Introduction

Triggered by the growing gap between suitable organs and patients on the waiting list, substantial effort has been made to expand the donor pools by implementing new technologies and procurement protocols. Cold storage preserves graft function by a reduction of cellular metabolism within a limited timeframe of ischemic time [1]. Cold ischemia is followed by reperfusion resulting in ischemia–reperfusion (I/R) injury [2]. Mitochondria are the essential primary target of I/R damage [3, 4]. After prolonged cold ischemia, reperfusion results in the decline of mitochondrial respiratory capacity decreasing the ability to generate adenosine triphosphate (ATP) [5, 6]. As myocardial ATP storages are depleted within seconds, myocytes are highly dependent on a well‐functioning mitochondrial respiratory system. Any defect leads to myocardial contractile dysfunction [7].

A possible way to reduce cold ischemia time is normothermic oxygenated *ex‐situ* perfusion (ESHP), which minimizes I/R injury and extends out of body time [8, 9]. Nevertheless, normothermic ESHP is limited by the functional decline over time, due to inflammatory cascades and oxidative stress related to metabolic perturbation [10, 11, 12].

Mitochondria represent a key target of interest when preserving or improving myocardial function in ESHP [13, 14]. Aim of this study was to investigate mitochondrial function during ESHP using high‐resolution respirometry (HRR) and to identify potential defects in the mitochondrial respiratory system [5, 6].

## 2 Materials and Methods

This study was approved by the Institutional Animal Care and Use Committee of the University Innsbruck and the Austrian Ministry of Science, Research and Economy (Nr. GZ 2020–0.363.115). All experiments were performed in compliance with European Union regulations for animal experimentation (Directive 2010/63/EU of the European Parliament and the European Council). ChatGPT was used to assist with language editing.

### 2.1 Animals and Heart Procurement

Six domestic pigs (50 to 70 kg) received intramuscular injection with azaperone (4 mg/kg; Jansen, Vienna, Austria) and atropine (0.01 mg/kg) 1 h preoperatively. After a single dose of ketamine (30 mg/kg) and propofol (1 mg/kg) intravenously, orotracheal intubation was established. Anesthesia was maintained with propofol (4–6 mg/kg/h) and remifentanil (0.2–0.3 μg/kg/min). After sternotomy 600 units (U)/kg of heparin were administered followed by activated clotting time measurement. After collection of 1000 mL whole blood, aortic cross clamping was performed and 1 L of cold (4°C) intracellular crystalloid cardioplegia (Custodiol HTK Solution, Dr. Franz Köhler GmbH, Bensheim, Germany) solution was administered. The explanted hearts were stored in cold saline during back table preparation. A standard 3/8″ connector (Euroset S.r.l., Medolla, Italy) was placed in the ascending aorta, a venous cannula (DLP Malleable Single stage venous cannulae, 30 Fr, Medtronic, Minneapolis, USA) in the pulmonary artery, a left ventricular vent (DLP 16Fr, Medtronic, Minneapolis, USA) was inserted via the left atrium into the left ventricle and pacemaker wires were placed.

### 2.2 *Ex‐Situ* Heart Perfusion

A self‐constructed Langendorff perfusion device was manufactured including a centrifugal pump (Revolution Ph.I.S.I.O, LivaNova, PLC, London, UK), venous reservoir and oxygenator (Paragon Pediatric PMP, Chalice Medical Ltd., Nottinghamshire, UK). The device was primed with 1000 mL leukocyte‐depleted whole blood, 500 mL of crystalloid fluid, 100 mL of albumin, 12.5 g of mannitol and a multivitamin preparation (Figure 1). Arterial perfusion was established by connecting the arterial line to the ascending aorta. The left ventricle was maintained in an unloaded, beating state and vented to prevent air embolism in the aortic root. The right atrium was closed, and a cannula was inserted into the pulmonary artery to enable measurement of coronary flow. Sequential perfusate measurements were performed for guidance of optimization in line with currently clinical practice [15]. Oxygen and gas flow were maintained with arterial partial pressure of oxygen (PaO2) of 100–200 mmHg and partial pressure of carbon dioxide (PaCO2) of 35–45 mmHg. Insulin, epineprine, and a combined glucose‐, aminoacid‐ and electrolyte substitution were continuously infused.

**FIGURE 1:**
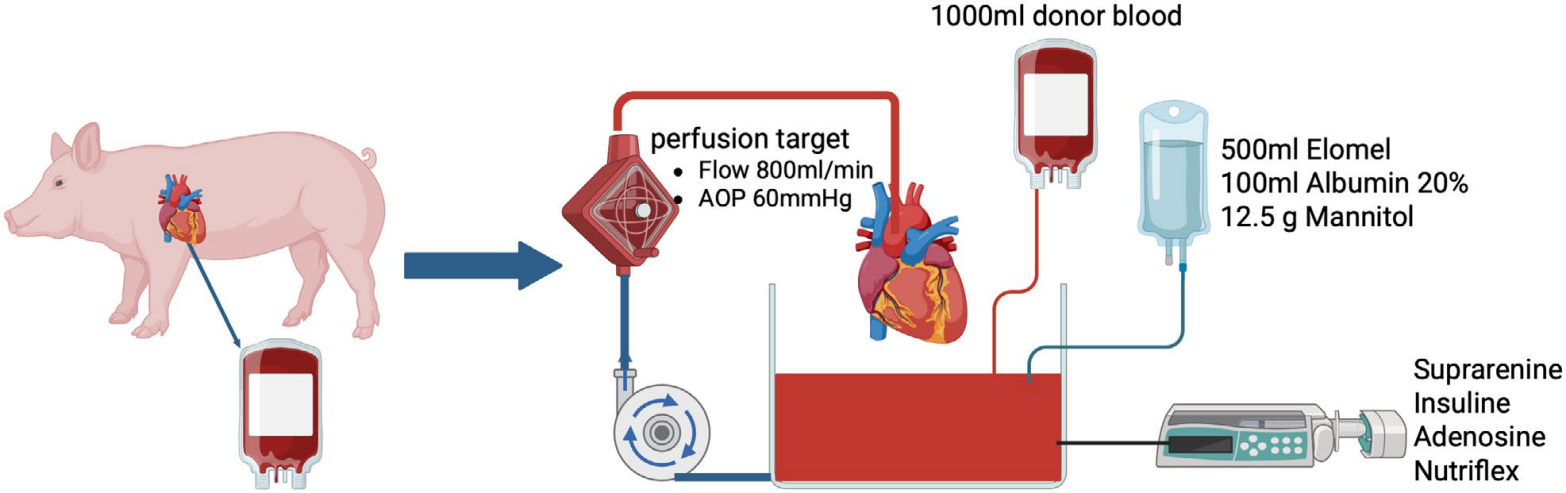
Schematic structure of the experiment.

Hearts were perfused for 6 h in Langendorff mode. Samples were collected before organ harvest in situ (starting sample), and during ESHP after 60, 180, and 360 min.

### 2.3 Myocardial Oxygen Consumption

Myocardial oxygen consumption (MVO_2_) was calculated by multiplying coronary blood flow (CBF) with the difference between arterial and venous oxygen content (Figure 2).

**FIGURE 2:**
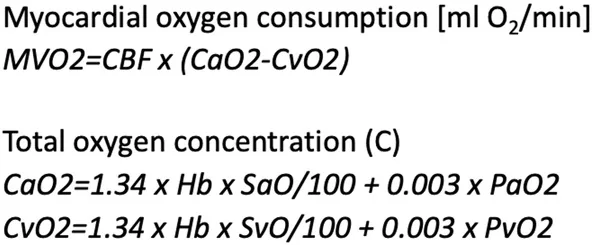
Calculation of myocardial oxygen consumption. CaO_2_, total oxygen concentration arterial (mL O_2_/mL blood); CBF, coronary blood flow (mL/min); CvO_2_, total oxygen concentration venous (mL O_2_/mL blood); Hb, hemoglobin (mg/dL); PaO_2_, arterial partial pressure of oxygen (mmHg); PvO_2_, venous partial pressure (mmHg); SaO, arterial oxygen saturation (%); SvO, venous oxygen saturation (%).

### 2.4 Myocardial Function

Cardiac performance was measured using a modified left ventricular pressure balloon (Ascendra catheter, Edwards Lifescience Corporation, Irvine, CA, USA) inflated in the left ventricle to achieve a preload of 15–20 mmHg during diastole 60, 180 and 360 min of ESHP [16, 17].

### 2.5 High‐Resolution Respirometry

Myocardial tissue specimens were obtained before organ harvest as baseline, 60, 180, and 360 min after ESHP from the left ventricular free wall with a 4 mm biopsy punch. Samples were stored in a mitochondrial respiration medium MiR05 (MiR05‐Kit, Oroboros Instruments GmbH, Innsbruck, Austria), consisting of 0.5 mM EGTA, 3 mM MgCl_2_ × 6 H_2_O, 60 mM lactobionic acid, 20 mM taurine, 10 mM KH_2_PO_4_, 20 mM HEPES, 110 mM sucrose, and 1 g/L of BSA (pH 7.1) on ice and transported directly to the cooling chamber (4°C) of the HRR facility.

#### 2.5.1 Preparation of Myocardial Tissue Homogenate

Ten milligrams cardiac muscle tissue was transferred to a shredder tube with 500 μL mitochondrial respiration medium MiR06 (MiR05 with the addition of 280 U/mL catalase). Samples were homogenized (PBI‐Shredder O2K‐Set, Oroboros Instruments GmbH, Innsbruck, Austria), placed on ice, and diluted with 9.5 mL of MiR06. Finally, the crude homogenate was filtered through a polyamide net filter with a loop size of 180 μm (Merck Millipore, Massachusetts, USA) and immediately transferred into the O2k chambers.

#### 2.5.2 High‐Resolution Respirometry Measurements

Mitochondrial respiratory function was assessed by HRR after calibration (O2k, Oroboros Instruments GmbH, Innsbruck, Austria) equipped with two 0.5 mL chambers for parallel measurements [18]. Dat lab software (DatLab version 7.4, Oroboros Instruments GmbH, Innsbruck, Austria) was used for analysis. MiR06 was selected as respiration medium as it contains catalase and therefore allows an increase in the oxygen levels in the measurement chambers by injection of hydrogen peroxide (50 mM, 1 μL) if required [19].

Two different substrate‐inhibitor‐uncoupler titration (SUIT) protocols were applied for investigation of NADH‐linked respiration in detail (Complex I, CI‐linked) respiration and OXPHOS state of fatty acid oxidation, NADH‐linked and succinate‐linked respiration. Measurements were performed simultaneously and in technical duplicates at 37°C, under constant stirring at 750 rpm and oxygen concentrations between 200 μM and 50 μM. Each titration step was performed after respiration reached a steady state. Respiration rates were expressed as pmol·s^−1^·mg wet mass^−1^. The time required for tissue processing and HRR analysis is approximately 1 h per sample set.

#### 2.5.3 HRR Protocols

SUIT protocols allow for investigation of different mitochondrial respiratory states. Three different substrate pathways were investigated in our HRR measurements: the NADH pathway (N‐pathway) linked respiration, feeding electrons to Complex I after the addition of substrates malate, glutamate, and pyruvate. The fatty acid oxidation pathway (F‐pathway), which was fueled in our protocol by octanoylcarnitine in the presence of low concentrations of malate. Finally, the succinate pathway (S‐pathway), which represents the respiration driven by the Complex II substrate succinate [18]. Details on SUIT protocols are depicted in Tables 1 and 2 and in Supporting Information and published data by Gnaiger et al. [19].

**TABLE 1:** Protocol overview of sequence of substrates and inhibitors of both protocols.

Protocol 1		
1.	Pyruvate	5 mM
2.	Malate	2 mM
3.	Glutamate	10 mM
4.	ADP	5 mM
5.	Cytochrome *c*	10 μM
6.	Octanoylcarnitine	0.5 mM
7.	CCCP	0.5 μM
8.	Antimycin A	2.5 μM

Protocol 2		
1.	ADP	5 mM
2.	Malate	0.1 mM
3.	Octanoylcarnitine	0.5 mM
4.	Cytochrome *c*	10 μM
5.	Malate	2 mM
6.	Pyruvate	5 mM
7.	Glutamate	10 mM
8.	Succinate	10 mM
9.	CCCP	0.5 μM
10.	Rotenone	0.5 μM
11.	Antimycin A	2.5 μM

**TABLE 2:** Key parameters and calculations in mitochondrial respiration protocols 1 and 2.

Protocol	Main purpose	Parameters	Calculation/ratio	Key insights
1	Investigate NADH‐linked (Complex I) respiration and mitochondrial coupling states	LEAK (L) respiration OXPHOS capacity (P) Cytochrome c response (Pc) Electron transfer capacity (E)	P–L control efficiency (1–L/P): ATP production efficiency Cytochrome c control efficiency (1–P/Pc): mitochondrial outer membrane integrity E–P control efficiency (1–P/E):phosphorylation system control	Evaluates efficiency of ATP production, mitochondrial membrane integrity, and phosphorylation control
2	Analyze substrate pathway control in OXPHOS (fatty acid, NADH‐, and succinate‐linked respiration)	OXPHOS (P) state of different substrate pathways	Flux control ratios (FCR): relative contributions of F‐, N‐, and S‐linked pathways to OXPHOS	Determines how different substrate pathways contribute to overall mitochondrial respiration

### 2.6 Statistical Analysis

Statistical analysis was done by GraphPad Prism 10 (GraphPad Software Inc., San Diego, CA, USA). All variables were assessed for normal distribution using the Shapiro–Wilk test. Given the small sample size, results were additionally verified by visual inspection of quantile‐quantile (Q‐Q) plots to ensure reliable interpretation of normality. All the data are presented as mean ± standard deviation. Normally distributed data are tested between two groups using a paired *t*‐test. Analysis of time‐dependent changes of various parameters was performed using repeated measurement ANOVA with Tukey's multiple comparisons test to define differences between single time points. If assumptions were violated, a Greenhouse–Geisser correction was performed. A *p*‐value of < 0.05 was considered statistically significant for all analyses.

## 3 Results

Hearts from 6 domestic pigs were perfused for 6 h in a customized Langendorff perfusion device. Baseline heart rate in donor pigs prior to organ procurement was 68 (±13) beats per minute under anesthesia. A stable heart rate was achieved by pacing the heart at 80 beats per minute during ESHP to standardize cardiac work across animals.

### 3.1 Metabolic Parameters

No significant change in arterial lactate levels during ESHP was found (starting sample 19 ± 12 mg/dL, 360 min 14 ± 5 mg/dL, F (2.576, 12.88) = 2.669; *p* = 0.0976) (Figure 3A).

**FIGURE 3:**
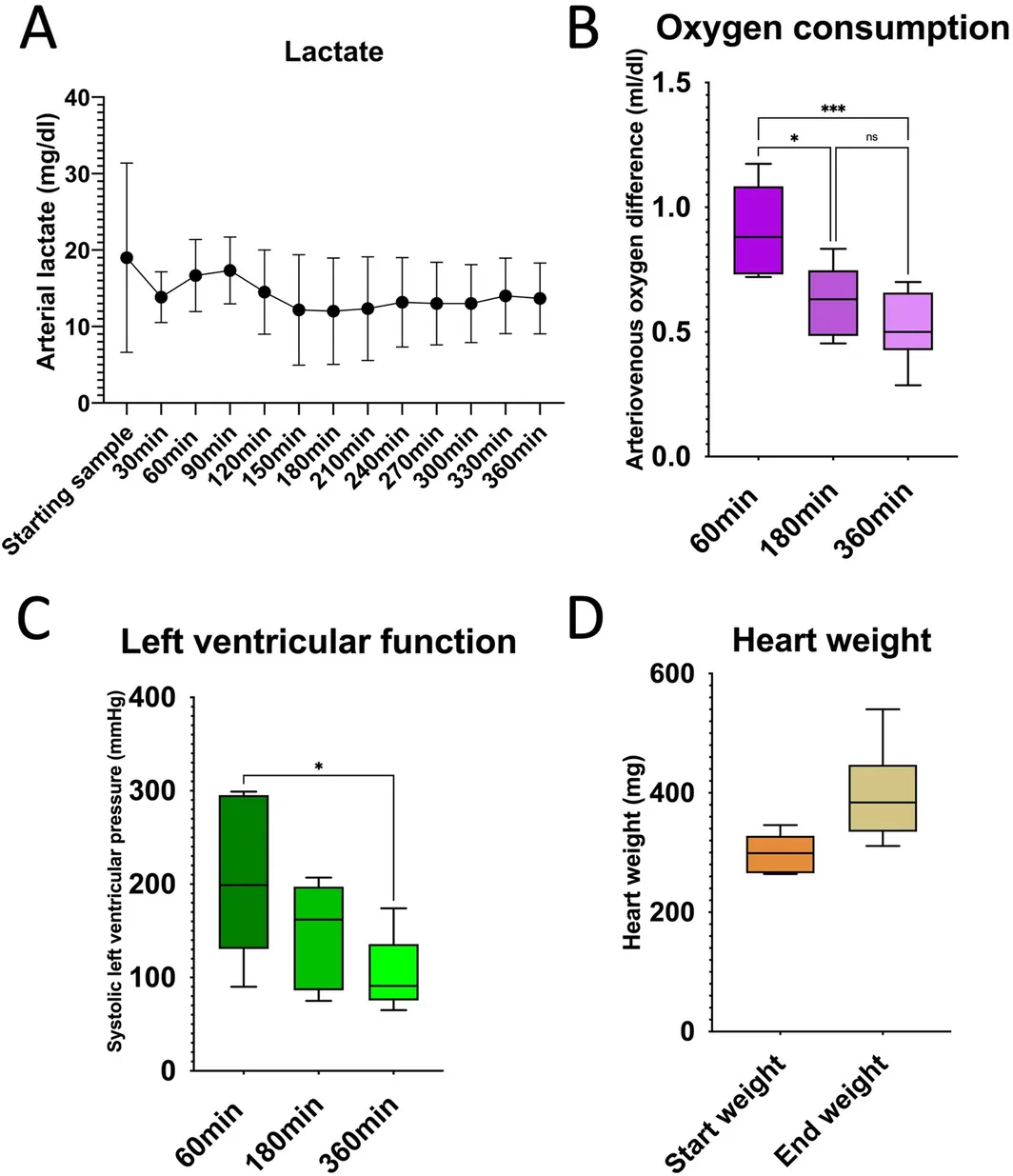
(A) Perfusate lactate levels, (B) myocardial oxygen consumption during ESHP, (C) left ventricular function, (D) myocardial edema formation.

Myocardial oxygen consumption decreased significantly (60 min 0.91 ± 0.18 mL/dL, 180 min 0.63 ± 0.14, 360 min 0.52 ± 0.15 mg/dL, (F (1.252, 6.262) = 34,76; *p* = 0.007)). A significant reduction was observed at 180 min (*p* = 0.0165), but values remained stable thereafter (*p* = 0.074) (Figure 3B).

### 3.2 Myocardial Function

Left ventricular function declined continuously over time (60 min 204 ± 83 mmHg, 180 min 148 ± 54 mmHg, 360 min 104 ± 40 mmHg, (F (1.795, 8.976) = 13.97; *p* = 0.002)) resulting in a significant difference between 60 min and end of perfusion at 360 min (*p* = 0.0119) (Figure 3C).

### 3.3 Edema Formation

Weight gain was significant, indicated the development of myocardial edema (start weight was 299.3 ± 33.5 mg, end weight of 396.3 ± 80.8 mg (*p* = 0.006)) (Figure 3D).

### 3.4 Mitochondrial Respiration During Ex‐Situ Heart Perfusion

#### 3.4.1 Pathway Control of Mitochondrial Respiration

Capacity of mitochondrial oxidative phosphorylation (*P*) for NADH‐, F‐ and succinate‐linked pathways in the baseline sample is depicted in Figure 4. Mass‐specific respiration was highest after addition of N‐pathway specific substrates (83.85 ± 33.19 pmol·s^−1^·mg wet mass^−1^), followed after the addition of succinate‐linked specific substrates (66.69 ± 10.41 pmol·s^−1^·mg wet mass^−1^) and finally fatty acid‐linked substrates (24.41 ± 11.57 pmol·s^−1^·mg wet mass^−1^) (F (1,064, 5322) = 18,51, *p* = 0.0064). Respiration after the addition of fatty acids was significantly lower than N‐pathway (*p* = 0.0041) and as S‐pathway (*p* = 0.0006).

**FIGURE 4:**
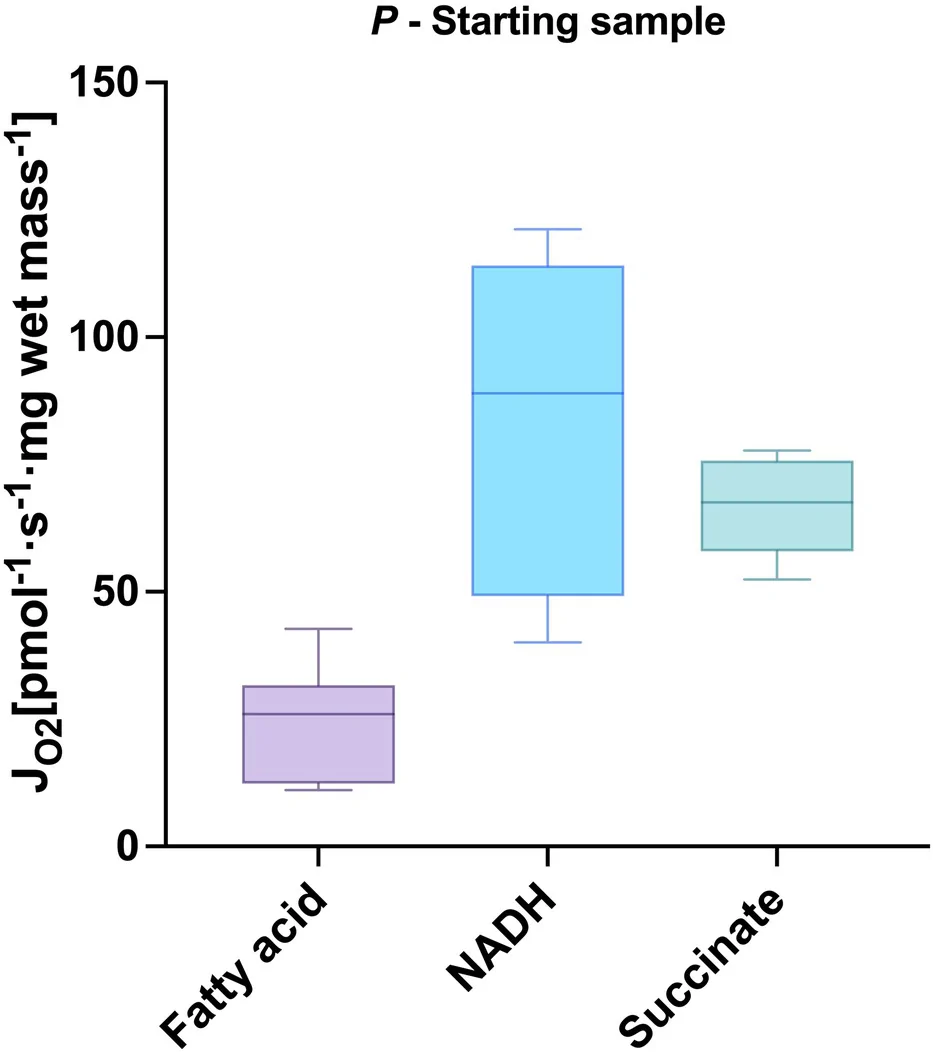
Oxidative phosphorylation capacity of starting sample according to substrate pathway–fatty acid oxidation pathway (F‐pathway), NADH pathway (N‐pathway) addition of specific substrates of complex I (malate, glutamate, pyruvate), succinate pathway (S‐pathway) respiration of Complex II by adding succinate.

Repeated HRR measurements showed stable F‐, N‐, and S‐linked OXPHOS capacities (P) (Figure 5 and Table 3).

**FIGURE 5:**
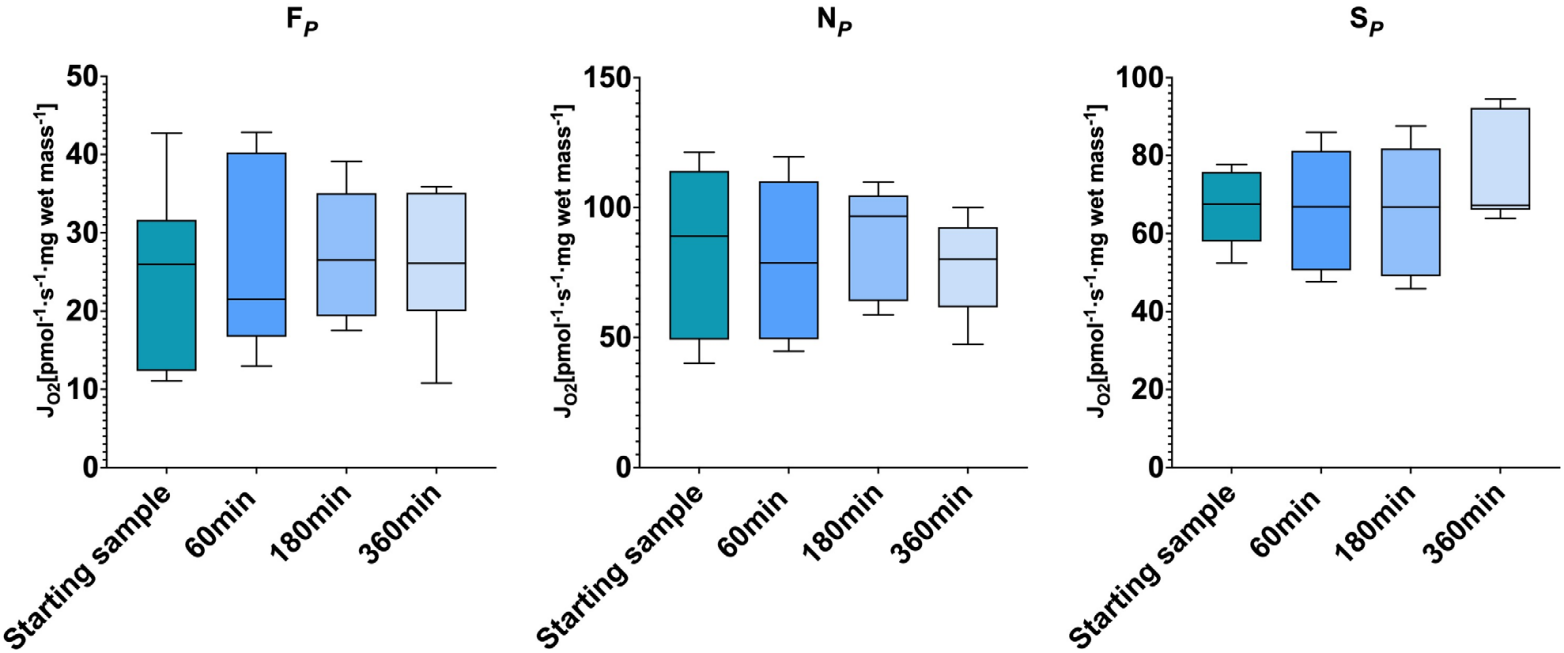
Oxidative phosphorylation capacity (P) of F‐, N‐, and S‐pathway.

**TABLE 3:** Summarized data F_P_, N_P_, and S_P_.

	Starting sample	60 min	180 min	360 min	One‐Way ANOVA
F_*P*_ (pmol·s^−1^·mg wet mass^−1^)	24.41 ± 11.57	26.03 ± 12.14	27.22 ± 8.794	26.13 ± 9.256	F (1.892, 9.462) = 0.07954; *p* = 0.9158
N_*P*_ (pmol·s^−1^·mg wet mass^−1^)	82.27 ± 37.13	81.49 ± 26.97	95.43 ± 23.23	90.35 ± 22.11	F (2.573, 12.86) = 0.1787; *p* = 0.8847
S_*P*_ (pmol·s^−1^·mg wet mass^−1^)	66.69 ± 10.41	66.41 ± 15.56	66.18 ± 17.27	75.2 ± 13.86	F (2.234, 11.17) = 0.5124; *p* = 0.6318

Flux control ratios (*FCR*) in relation to the respiration were measured in every single substrate pathway for convergent electron transfer in OXPHOS state (P_FNS_) (Figure 6). The time course remained stable (F (3, 60) = 0.000, *p* > 0.999), while a significant difference was observed between the pathways (F (2, 60) = 198, *p* < 0.0001). NADH‐linked *FCR* decreased at 360 min, whereas succinate‐linked *FCR* increased resulting in a similar ratio of both pathways without any significance. (*p* = 0.9999) (Table 4).

**FIGURE 6:**
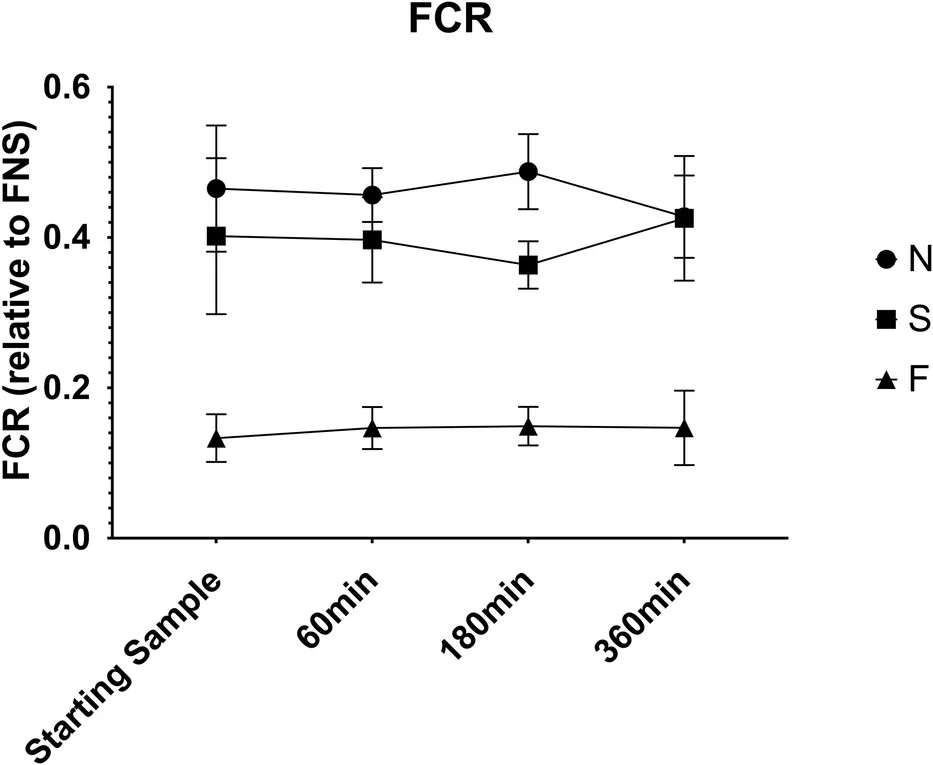
Flux control ratio (FCR) of N‐, S‐, and F‐pathway in relation to oxidative phosphorylation capacity (FNS_P_).

**TABLE 4:** Summarized data FCR of N‐, S‐, and F‐pathway.

	Starting sample	60 min	180 min	360 min	One‐way ANOVA
FCR F	0.13 ± 0.03	0.15 ± 0.03	0.15 ± 0.03	0.15 ± 0.05	F (2,179, 10,89) = 0,2991; *p* = 0.7648
FCR N	0.47 ± 0.08	0.46 ± 0.04	0.49 ± 0.05	0.43 ± 0.05	F (1,577, 7885) = 1129; *p* = 0.3545
FCR S	0.40 ± 0.10	0.40 ± 0.06	0.36 ± 0.03	0.43 ± 0.08	F (1,484, 7421) = 0,6772; *p* = 0.4943

#### 3.4.2 Phosphorylation Efficiency and Capacity

Leak respiration (Figure 7A) analyzed by applying SUIT Protocol 1 did not change during ESHP (starting sample 9.56 ± 4.47 pmol·s^−1^·mg wet mass^−1^, 360 min 12.92 ± 3.58 pmol·s^−1^·mg wet mass^−1^; F (2.413, 12.06) = 0.9301, *p* = 0.4376). There was also no change in *P*_*N*_ *‐L*_*N*_ control efficiency (Figure 7B) before and during the ESHP (starting sample 0.88 ± 0.03, 360 min 0.85 ± 0.05; F (1.544, 7.718) = 2.141; *p* = 0.1841).

**FIGURE 7:**
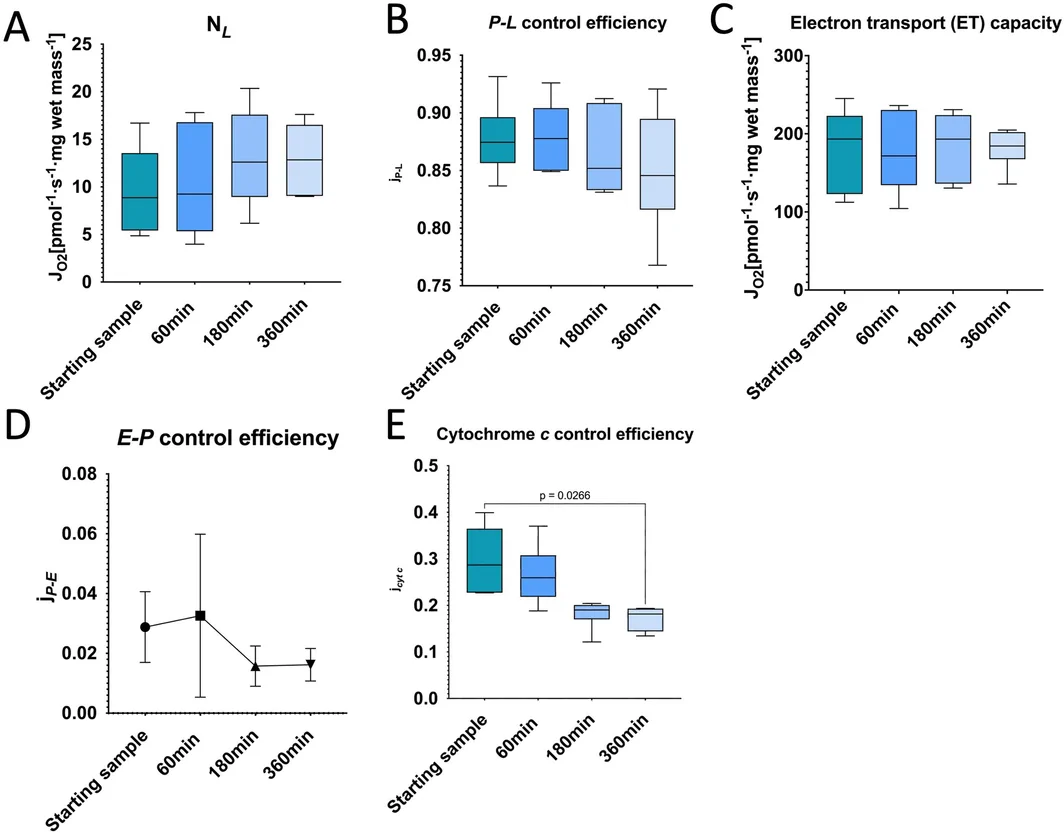
(A) N_L_—Leak respiration of NADH‐linked substrates; (B) P‐L control efficiency—calculated in N‐linked leak state and N‐linked Oxphos capacity; (C) Electron transport capacity; (D) E‐P control efficiency; (E) Cytochrome c control efficiency.

Stepwise titration of CCCP investigated electron transport (ET) capacity. Figure 7C shows a stable respiration after uncoupling titrations (starting sample 181 ± 52.12 pmol·s^−1^·mg wet mass^−1^, 360 min 181.4 ± 24.8 pmol·s^−1^·mg wet mass^−1^; F (2,418, 12,09) = 0,05459, *p* = 0.9670). To evaluate possible control of the phosphorylation system over the oxidation system, E‐P control efficiency (Figure 7D) was calculated (1‐*P*/*E*). The ratio did not change in the time course (starting sample 0.029 ± 0.029, 360 min 0.016 ± 0.013; F (1.392, 6.958) = 0.3576, *p* = 0.6384).

#### 3.4.3 Outer Mitochondrial Membrane Damage (mtOM)

The cytochrome *c* control efficiency (1‐*P*/*P*_c_) of the NADH‐pathway (Figure 7E) showed a slight decrease during the time course (starting sample 0.30 ± 0.07, 60 min 0.27 ± 0.06, 180 min 0.18 ± 0.03, 360 min 0.17 ± 0.03; F (1.670, 8.348) = 8346; *p* = 0.1841), reaching significance after 360 min of ESHP (*p* = 0.0266).

## 4 Discussion

Characterization of myocardial and mitochondrial function during 6 h of normothermic ESHP using HRR in a porcine model revealed the following results: ESHP resulted in a significant decrease in myocardial function and myocardial oxygen consumption. HRR revealed preserved oxidative phosphorylation capacities in NADH‐, succinate‐, and fatty acid oxidation–linked pathways during 6 h of ESHP. Phosphorylation efficiency and electron transport capacity were unchanged, indicating that mitochondrial ATP production and coupling efficiency were maintained.

While normothermic ESHP can extend the out‐of‐body time of donor hearts and expand the donor pool [20, 21], the technology is accompanied by limitations. Although lactate trends are capable of guiding perfusion management, valid parameters for organ quality and predictive capacity are lacking [22]. We were able to characterize tissue bioenergetics during normothermic ESHP and install HRR as a promising evaluation tool for guided optimization of ESHP.

HRR enables insights into the efficiency and possible dysfunctions of mitochondrial respiration. In previous studies, HRR could demonstrate an impaired oxidative phosphorylation capacity in heart failure [23], in cardiac allograft vasculopathy [24] and in heart transplant rejection [25]. Findings from cold stored rat hearts suggest a strong correlation between graft function and mitochondrial respiratory capacity [6]. In our investigation, HRR results showed stable oxidative phosphorylation capacities across the NADH‐, fatty acid oxidation‐, and succinate‐linked pathways during 6 h of ESHP. NADH‐linked respiration was slightly higher than succinate‐linked and even four times higher than fatty acid oxidation (FAO)‐linked respiration in the starting sample. This result differs from previous reports in mouse and human myocardium, where succinate‐linked respiration was significantly higher [26, 27].

After 6 h of perfusion, a subtle shift in mitochondrial respiratory patterns was observed. The calculated flux control ratio indicated a decrease in NADH‐linked respiration alongside a relative increase in succinate‐linked respiration, resulting in an overall comparable ratio. This finding may suggest that the duration of perfusion is intrinsically limited, with early alterations in substrate utilization preceding overt mitochondrial dysfunction [6].

Berg et al. investigated mitochondrial function in hypothermic oxygenated machine perfusion in a porcine donation after circulatory death (DCD) model; they reported a decline in overall oxidative phosphorylation (OXPHOS) capacity [28]. In line with this, Seefeldt et al. examined mitochondrial function following normothermic regional perfusion in a DCD porcine model and also observed a reduction in OXPHOS capacity, primarily driven by diminished NADH‐linked respiration [29].

Together, these findings support the notion that NADH‐linked respiration represents a particularly vulnerable component of the electron transport system, potentially serving as an early indicator of mitochondrial impairment under prolonged perfusion conditions.

Damage to the mitochondrial outer membrane leads to cytochrome c release—potentially limiting mitochondrial oxidative phosphorylation. This condition can be partially reversed with addition of external cytochrome c during HRR measurements [30]. Thus, an increase in respiration after cytochrome *c* addition indicates impaired integrity of the outer mitochondrial membrane. Interestingly, we observed a slight decrease in cytochrome *c* control efficiency. This finding translates into a decline in the extent of the damage of the outer mitochondrial membrane—a very promising finding in terms of mitochondrial recovery during ESHP.

The preservation of high P‐L control efficiency and low E‐P control efficiency ratios throughout the perfusion suggests that ATP production remained stable during ESHP and that oxidative phosphorylation was not limited by the phosphorylation system. This stability is particularly important for the heart as it reflects an efficient mitochondrial function and ATP production.

Myocardial oxygen consumption (MVO_2_) decreased after the first hour, which could be interpreted as an initial high metabolic demand that stabilizes as the heart adapts to the ESHP conditions.

In contrast to previously published studies on chronic disease models, which report an association between mitochondrial dysfunction and impaired cardiac performance [23, 24, 25], our findings indicate that graft function declined despite preserved mitochondrial function. This suggests that mitochondrial impairment is unlikely to be the primary driver of functional deterioration in our model. Specifically, we were able to exclude insufficient ATP production due to mitochondrial damage as a key mechanism. Instead, we propose that the observed reduction in left ventricular (LV) function and myocardial oxygen consumption (MVO_2_) during ex situ heart perfusion (ESHP) may be attributable to extramitochondrial factors, including alterations in perfusate composition, progressive myocardial edema, or depletion of essential metabolic substrates, rather than intrinsic mitochondrial failure.

Our finding of the decline in ventricular function, and the increase in heart weight aligns with findings from other studies, suggesting a consistent challenge in the preservation of heart function under normothermic conditions [31, 32]. The absence of a clearance or elimination mechanism during standard ESHP may lead to the accumulation of metabolic byproducts or imbalances in the perfusate, thereby adversely affecting myocardial performance. Notably, Tchouta et al. [12] demonstrated that prolonged ESHP could be achieved through continuous plasma exchange of the perfusion fluid. Together with recent advances in cardioprotective perfusion strategies, these findings underscore the importance of a multifaceted approach to optimizing heart preservation techniques [33, 34, 35, 36].

Hypothermic oxygenated machine perfusion (HOPE) has shown promising results as an alternative preservation strategy [37]. Notably, See Hoe et al. recently demonstrated in a transplanted ovine model that HOPE improved cardiac mitochondrial function compared to static cold storage [38]. Comparing mitochondrial respiratory profiles during HOPE and normothermic ESHP using HRR would be a valuable direction for future research, particularly in the context of marginal donor hearts, extended preservation times, and donation after circulatory death, where mitochondrial vulnerability is substantially greater.

## 5 Limitations

Limitations of this study include the absence of a designated control group. However, measurements were conducted on the beating porcine heart in situ before organ harvest, serving as a reference for a healthy, non‐perfused heart, as well as at multiple time points during ex situ heart perfusion. Brain death or circulatory death simulation was not performed. Therefore, the influence of these modalities on ex‐situ heart perfusion and HRR were not investigated. The absence of these stressors in our model likely contributed to the well‐preserved baseline and sustained mitochondrial function observed throughout ESHP. High‐resolution respirometry is a sensitive, but also technically advanced method. To reduce inter‐user variability and technical variability all analysis were performed by one expert in addition to performance of technical and biological replicates, strict standardization of the technique and quality control measures. The current study does not provide clinically applicable cut off values; Normothermic ESHP was performed according to the standard protocols currently used in clinical routine, still the device was custom made with substantial room for improvement (e.g., hemocompatibility of tubing and pump, lack of sterility). Potential confounding effects on HRR results cannot be fully excluded, as not all clinically relevant infusion and perfusion variables can be assessed within this single experiment. The balloon‐based functional measurement provides an approximation of systolic performance under fixed preload but cannot fully characterize left ventricular mechanics.

## 6 Conclusion

Our findings characterize the preservation of mitochondrial function, the essential powerhouse to maintain the heart's energy reserves, during normothermic ESHP. The technology of HRR could act as a fast and real‐life evaluation tool for mitochondrial assessment, providing a deeper understanding of molecular processes during ESHP. Implementing HRR in standard organ evaluation protocols could help to better characterize organ quality during ESHP. Future studies focusing on the characterization of HRR measurements in a DCD setting are needed as the next step.

## Author Contributions

L.S.: Concept, data analysis and interpretation, drafting article, statistics; J.H.: Concept, data analysis, revision of article; A.T.M.: Concept, data analysis, revision of article; A.A.: Data collection; G.P.: Concept, data collection; N.P.: Data collection; N.H.: Data collection; F.S.: Concept, data collection; J.M.: Concept, data collection; J.W.: Data collection; M.G.: Supervision; S.S.: Supervision; J.D.: Concept, data collection, revision and approval of article, funding.

## Funding

This work was supported by Tiroler Wissenschaftsförderung, F.16942/5‐2019. Society for the Promotion of Cardiovascular Research.

## Conflicts of Interest

The authors declare no conflicts of interest.

## Supporting information


**Data S1:** aor70213‐sup‐0001‐DataS1.docx.

## Data Availability

The data that support the findings of this study are available on request from the corresponding author. The data are not publicly available due to privacy or ethical restrictions.
