# Supplementary material for: Monitoring Mitochondrial Function With High‐Resolution Respirometry During Ex‐Situ Heart Perfusion
Source: Artif Organs. 2026 Sep 10;50(11):1575–84. doi: 10.1111/aor.70213 (PMC13720659; doi:10.1111/aor.70213)
Supplement: Supplementary file 1 [file AOR-50-1575-s001.docx]

Supplemental material

#### Substrate – uncoupler – inhibitor tritration protocols

| Protocol 1 | |  |  | Protocol 2 | |  |
| --- | --- | --- | --- | --- | --- | --- |
| 1. | Pyruvate | 5 mM |  | **1.** | ADP | 5 mM |
| 2. | Malate | 2 mM |  | **2.** | Malate | 0.1mM |
| 3. | Glutamate | 10mM |  | **3.** | Octanoylcarnitine | 0.5mM |
| 4. | ADP | 5mM |  | **4.** | Cytochrome C | 10µM |
| 5. | Cytochrome C | 10µM |  | **5.** | Malate | 2mM |
| 6. | Octanylcarnitine | 0.5mM |  | **6.** | Pyruvate | 5mM |
| 7. | FCCP | 0.5µM |  | **7.** | Glutamate | 10mM |
| 8. | Antimycin A | 2.5µM |  | **8.** | Succinate | 10mM |
|  |  |  |  | **9.** | FCCP | 0.5µM |
|  |  |  |  | **10.** | Rotenone | 0.5µM |
|  |  |  |  | **11.** | Antimycin a | 2.5µM |

#### HRR Protocols

Using substrate-uncoupler-inhibitor titration protocols allows to investigate different respiratory states of the mitochondria. The stepwise addition of different substituents of the respiratory chain allows to measure respiratory pathways individually.

Three different substrate pathways are investigated in our protocols:

NADH pathway (N-pathway) linked respiration after the addition of the specific substrates of complex I. These include malate, glutamate, and pyruvate.

The fatty acid oxidation pathway (F-pathway), which is characterized by the substrate octanoylcarnitine.

And finally, the succinate pathway (S-pathway), which represent the respiration of Complex II by adding succinate. Together with the addition of adenosine-diphosphate (ADP), the measurement shows how effective oxidative phosphorylation in the mitochondria works.

Additionally, HRR is able to measure the integrity of the outer mitochondrial membrane by adding cytochrome c.

Finally, repeated titration of Carbonyl cyanide-4 (trifluoromethoxy) phenylhydrazone (CCCP) is titrated until the maximum respiratory rate, measuring full uncoupling to evaluate the electron transfer (E) capacity .

Protocol 1 was designed to investigate NADH-linked (or Complex I-, CI-linked) respiration in detail, including different mitochondrial coupling states. Non-phosphorylating LEAK (*L*) respiration was reached after the addition of pyruvate, malate and glutamate. Then, adenosine-diphosphate (ADP) was added to analyse how effective the oxidative phosphorylation works. This process is called OXPHOS capacity (*P*). Subsequent, cytochrome *c* (*P_c_*) is added to evaluate mitochondrial outer membrane integrity. Finally, carbonyl cyanide p-trifluoromethoxyphenyl hydrazone (CCCP) was titrated step-wise until maximum respiration was reached to assess the electron transfer (ET) capacity. Calculation of *P-L* control efficiency was performed (1-*L*/*P*), which allows the evaluation of efficiency of ATP production. Furthermore, cytochrome *c* control efficiency (1-*P*/*P*_c_) was calculated to evaluate the integrity of the mitochondrial outer membrane. Finally, *E-P* control efficiency (1-*P*/*E*) was calculated to investigate if the phosphorylation system exerts control over the OXPHOS capacity

Protocol 2 enables analysis of substrate pathway control in the OXPHOS state of fatty acid oxidation, NADH-linked and succinate-linked respiration. OXPHOS (*P*) state with saturating concentrations of ADP and pathways specific substrates were investigated. Additionally, ET capacity was analysed after the stepwise addition of CCCP. Relative contributions of the three substrate pathways, expressed as flux control ratios (*FCR*), were calculated in relation to OXPHOS capacity of F-, N- and S-linked respiration.

*Respiratory states*
There are different respiratory states and coupling states in high resolution respirometry. To give an overview, we will describe the most important states.

Oxphos capacity (P)
Oxidative phosphorylation is the oxidation of energy-rich substances by electron transfer to oxygen. This process is coupled to phosphorylation of ADP to ATP. Thus, the OXPHOS capacity describes the respiratory capacity with ADP, saturating concentrations of substrates, oxygen and phosphate.

LEAK (L)
Leak respiration is measured when the substrates are added but no ADP is present. This reflects the proton gap, which consumes oxygen and produces heat, but is not involved in ATP production.

Electron transfer (E) capacity
The electron transfer capacity is the maximum possible rate of electrons that can be transferred from complex to complex in the uncoupled state. Therefore, this capacity is independent of the maximum phosphorylation capacity.

P-L control efficiency (1-L/P)

This ratio describes the correlation between leak and oxidative phosphorylation, which allows for evaluation of the efficiency of ATP production.

E-P control efficiency (1-P/E)
This ratio is calculated to evaluate the limitation of the oxidative phosphorylation capacity by the phosphorylation system

Cytochrome c control efficiency (1-P/ Pc)
Cytochrome c is a component of the respiratory chain. It transfers electrons from complex 3 to 4. Cytochrome c is not able to pass through an unscathed outer mitochondrial membrane. Therefore, in the case of a damage of the outer mitochondrial membrane, cytochrome c is able to pass and detectable.

Flux control ratios (FCR)
FCR are proportions of oxygen flow in various states standardised to the maximum flow in shared reference state. The maximum flow was achieved in the uncoupled state.
